# Supplementary material for: The Role of Indocyanine Green Fluorescence in Rectal Cancer Robotic Surgery: A Narrative Review
Source: Cancers (Basel). 2022 May 13;14(10):2411. doi: 10.3390/cancers14102411 (PMC9139806; doi:10.3390/cancers14102411)
Supplement: Supplementary file 1 [file cancers-14-02411-s001.zip › cancers-1701701-supplementary.pdf]

Supplementary materials

Table S1. Critical appraisal of included studies using Newcastle Ottawa scale. **Stars number**

**Study, year**	Selection[a]	Comparability[b]	Outcome[c]	Total
**Jafari, 2013**	****	**	***	9
**Bae, 2015**	***	**	***	8
**Kim, 2019**	****	**	***	9
**Somashekhar, 2020**	***	*	***	7

[a] Maximum 4 stars; [b] Maximum 2 stars; [c] Maximum 3 stars.
